# SARS-CoV-2 RBD and Its Variants Can Induce Platelet Activation and Clearance: Implications for Antibody Therapy and Vaccinations against COVID-19

**DOI:** 10.34133/research.0124

**Published:** 2023-04-24

**Authors:** Xiaoying Ma, Jady Liang, Guangheng Zhu, Preeti Bhoria, Aron A. Shoara, Daniel T. MacKeigan, Christopher J. Khoury, Sladjana Slavkovic, Lisha Lin, Danielle Karakas, Ziyan Chen, Viktor Prifti, Zhenze Liu, Chuanbin Shen, Yuchong Li, Cheng Zhang, Jiayu Dou, Zack Rousseau, Jiamin Zhang, Tiffany Ni, Xi Lei, Pingguo Chen, Xiaoyu Wu, Hamed Shaykhalishahi, Samira Mubareka, Kim A. Connelly, Haibo Zhang, Ori Rotstein, Heyu Ni

**Affiliations:** ^1^ Department of Laboratory Medicine and Pathobiology, University of Toronto, Toronto, ON, Canada.; ^2^ Department of Laboratory Medicine, Keenan Research Centre for Biomedical Science of St. Michael’s Hospital, Toronto, ON, Canada.; ^3^ Toronto Platelet Immunobiology Group, Toronto, ON, Canada.; ^4^ Department of Physiology, University of Toronto, Toronto, ON, Canada.; ^5^ CCOA Therapeutics Inc., Toronto, ON, Canada.; ^6^ Canadian Blood Services Centre for Innovation, Toronto, ON, Canada.; ^7^ The State Key Laboratory of Respiratory Disease, Guangzhou Institute of Respiratory Disease, The First Affiliated Hospital of Guangzhou Medical University, Guangzhou, Guangdong, China.; ^8^ Department of Laboratory Medicine, The Second Affiliated Hospital of Guangzhou University of Chinese Medicine, Guangzhou, China.; ^9^ Advanced Pharmaceutics & Drug Delivery Laboratory, Leslie Dan Faculty of Pharmacy, University of Toronto, Toronto, ON, Canada.; ^10^ Department of Medical Microbiology and Infectious Disease, Sunnybrook Health Science Centre, Toronto, ON, Canada.; ^11^ Department of Medicine, University of Toronto, Toronto, ON, Canada.; ^12^ Division of Cardiology, St. Michael's Hospital, Toronto, ON, Canada.; ^13^ Department of Anesthesiology and Pain Medicine and Division of Critical Care Medicine, University of Toronto, Toronto, ON, Canada.; ^14^ Interdepartmental Division of Critical Care Medicine, University of Toronto, Toronto, ON, Canada.; ^15^ Department of Surgery, University of Toronto, Toronto, ON, Canada.

## Abstract

The COVID-19 pandemic caused by SARS-CoV-2 virus is an ongoing global health burden. Severe cases of COVID-19 and the rare cases of COVID-19 vaccine-induced-thrombotic-thrombocytopenia (VITT) are both associated with thrombosis and thrombocytopenia; however, the underlying mechanisms remain inadequately understood. Both infection and vaccination utilize the spike protein receptor-binding domain (RBD) of SARS-CoV-2. We found that intravenous injection of recombinant RBD caused significant platelet clearance in mice. Further investigation revealed the RBD could bind platelets, cause platelet activation, and potentiate platelet aggregation, which was exacerbated in the Delta and Kappa variants. The RBD–platelet interaction was partially dependent on the β3 integrin as binding was significantly reduced in β3^−/−^ mice. Furthermore, RBD binding to human and mouse platelets was significantly reduced with related αIIbβ3 antagonists and mutation of the RGD (arginine-glycine-aspartate) integrin binding motif to RGE (arginine-glycine-glutamate). We developed anti-RBD polyclonal and several monoclonal antibodies (mAbs) and identified 4F2 and 4H12 for their potent dual inhibition of RBD-induced platelet activation, aggregation, and clearance in vivo, and SARS-CoV-2 infection and replication in Vero E6 cells. Our data show that the RBD can bind platelets partially though αIIbβ3 and induce platelet activation and clearance, which may contribute to thrombosis and thrombocytopenia observed in COVID-19 and VITT. Our newly developed mAbs 4F2 and 4H12 have potential not only for diagnosis of SARS-CoV-2 virus antigen but also importantly for therapy against COVID-19.

## Introduction

Coronavirus disease 2019 (COVID-19) caused by severe acute respiratory syndrome coronavirus 2 (SARS-CoV-2) has reached to >651 million cases and has caused >6.6 million deaths as of December 2022. Despite its respiratory origin, systemic thromboembolic complications are a common feature of critically ill patients [1–3]. Pulmonary embolism [4], stroke [5], and myocardial infarction [6] are often reported complications of hospitalized patients. COVID-19 also continues to birth new variants that modulate associated cardiovascular complications. The Delta variant in particular has been linked with a higher risk of mortality, intensive care unit admission, hospitalization [7–9], and more serious blood clots [10,11]. However, the role of platelets in COVID-19 thrombosis and the effect of its variants have not been adequately explored.

Platelets are small anucleate blood cells known for their critical roles in thrombosis and hemostasis in mammals [12–16]. The life spans of platelets are relatively short, around 7 to 10 d in humans and 4 to 5 d in mice. Platelets are removed from circulation by the reticuloendothelial system, in which autoantibody-targeted platelets are predominantly cleared in the spleen, while aged/desialylated platelets are likely cleared in the liver by Kupffer cells [17–22]. Notably, hospitalized COVID-19 patients generally present with hyperactive platelets that may account for associated thrombosis and disseminated intravascular coagulation [23–25]. Platelet hyperactivity can also translate to consumption and clearance causing thrombocytopenia [17,26,27], a complication also reported in COVID-19 patients and is associated with a worse prognosis [28–30]. Interestingly, COVID-19 vaccinations that utilize the spike protein receptor-binding domain (RBD) have also been associated with reports of thrombosis and thrombocytopenia, known as vaccine-induced thrombotic thrombocytopenia (VITT) [31–35]. However, the platelet clearance mechanism in both COVID-19 and VITT remains unclear. Understanding the complexity of platelet hyperactivity and activation in COVID-19 and VITT is vitally important to help guide prophylactic efforts and treatment for critically ill patients.

There are some reports [36–38] that SARS-CoV-2 directly interacts with platelets, but the mechanism has not been well explored. Although angiotensin-converting enzyme-2 (ACE2) is the dominant receptor of the spike protein RBD, the expression of ACE2 on platelets remains controversial [23,25,36,37,39–41], perhaps in part due to genetic differences among patients and varying methodologies. Nevertheless, SARS-CoV-2–platelets interactions independent of ACE2 have been reported [37,41], indicating alternative cognate receptors [42–46]. Interestingly, the SARS-CoV-2 spike protein RBD contains the integrin-binding RGD (arginine-glycine-aspartate) motif that is absent in other coronaviruses [46–48].

Integrins are a large family of adhesion molecules, and 24 integrins have been identified [49,50]. There are 2 reported β3-containing integrins, αIIbβ3 and αVβ3. Different from αVβ3 integrin that might be expressed on various cells, αIIbβ3 is almost exclusively expressed on platelets and their precursor megakaryocytes. The αIIbβ3 receptor is the dominant β3-containing integrin expressed on platelets (~80,000 copies per platelet), compared to negligible quantities of αVβ3 integrin [50,51]. Structural and functional analyses of αIIbβ3 in human and some vertebrates show significant conservation in human and mouse. The primary structure of αIIbβ3 in human (UniProt IDs: αIIb, P08514; β3, P05106) and mouse (UniProt IDs: αIIb, Q9QUM0; β3, O54890) are 80.4% and 90.3% homologous, respectively [49,52]. Some studies reported that the RGD motif in the RBD can bind to integrins αVβ3 and αVβ6 and inconsistent findings concerning α5β1 [53–55]. In silico sequence analysis also suggests a potential interaction with integrin αIIbβ3 [46,47]; however, to our knowledge, there is no solid report that provides direct evidence for this interaction.

In the present study, we discovered that the SARS-CoV-2 spike protein RBD can bind platelets partially through integrin αIIbβ3 (GPIIbIIIa) and cause platelet activation and clearance, providing novel mechanisms of thrombosis and thrombocytopenia in COVID-19 and VITT.

## Results

### The RBD of the spike protein can induce platelet clearance in vivo

**Fig. 1.**
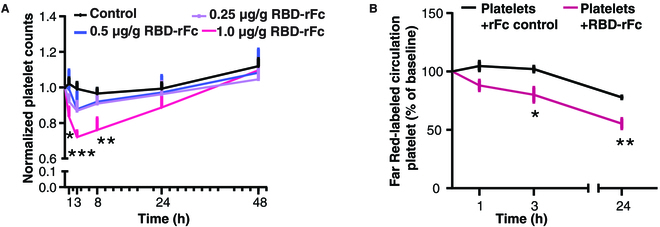
The RBD-rFc induces platelet clearance in vivo*.* (A) CD1 mouse platelets were counted at different time intervals (0, 1, 3, 8, 24, and 48 h) after I.V. injection with either a PBS control or RBD-rFc (PBS, *n* = 8; 0.25 μg/g RBD-rFc, *n* = 4; 0.5 μg/g RBD-rFc, *n* = 4; 1 μg/g RBD-rFc, *n* = 11). (B) A total of 2 × 10^8^ Far Red-labeled platelets were incubated with 2.5 μg/ml RBD-rFc for 30 min and then I.V. injected into CD1 mice. Circulating platelets were quantified at different time intervals (0, 1, 3, and 24 h). All the data were expressed as means ± SEM. Two-way ANOVA followed by Tukey’s multiple comparisons test was applied to evaluate the difference between RBD group and control group. *P < 0.05, **P < 0.01, ***P < 0.001, versus control at the same time point. *P < 0.05, **P < 0.01, versus baseline.

Since thrombocytopenia is associated with severe cases of COVID-19 and VITT, we first explored whether the RBD could induce platelet clearance in vivo. We generated recombinant RBD in a mammalian cell culture system linked with a rabbit Fc tag (RBD-rFc) for optimal stability and expression. We then intravenously injected different doses (0.25, 0.5, and 1.0 μg/g) of RBD-rFc into 6-week-old female CD1 mice and recorded the platelet counts at 0, 1, 3, 8, 24, and 48 h postinjection as we did for murine models of thrombocytopenia [17,56,57]. Platelet counts at the 1st, 3rd, and 8th hour after injection of 1.0 μg/g RBD-rFc decreased significantly and was most pronounced at the 3rd hour (RBD versus control, 1 h: *P < 0.05; 3 h: ***P < 0.001; 8 h: **P < 0.01). Conversely, the lower doses of 0.25 μg/g and 0.5 μg/g did not significantly (only trends) decrease platelet counts, indicating that lower doses of RBD were not sufficient to cause thrombocytopenia (Fig. 1A and Fig. S1A).

To confirm that the RBD induced platelet clearance instead of platelet sequestration, labeled mouse platelets were incubated with RBD-rFc for 30 min, then intravenously (I.V.) injected into CD1 mice, and quantified at 0, 1, 3, and 24 h posttransfusion. We found the RBD caused clearance of transfused platelet that did not recover up to 24 h posttransfusion (Fig. 1B), confirming that the drop in platelet count was due to clearance rather than sequestration. To exclude the possibility that the decrease in mouse platelet count was caused by the rabbit Fc tag attached to the RBD, RBD without a tag was generated after tobacco etch virus (TEV) protease cleavage of an N-terminal His-tag. Even after removal of the Fc tag, RBD-induced thrombocytopenia persisted, especially at 1 and 3 h postinjection (RBD versus control, 1 h: *P < 0.05; 3 h: **P < 0.01) (Fig. S1B).

### The SARS-CoV-2 spike protein RBD binds platelet β3 integrin

**Fig. 2.**
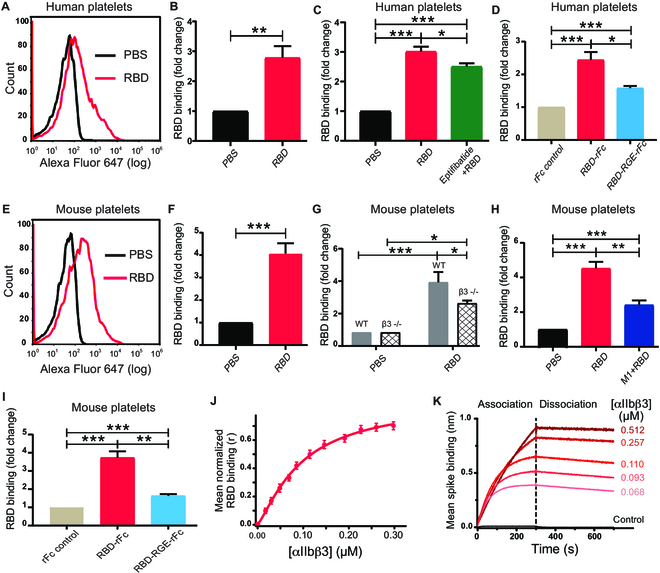
The RBD can bind to platelets partially via integrin αIIbβ3. The binding of RBD (10 μg/ml) to healthy human donor (A to C) and mouse (E to H) washed platelets were analyzed by flow cytometry using Alexa Fluor 647-labeled anti-RBD mAb 4H12. The binding of RBD-rFC to human (D) and mouse (I) platelets was analyzed by flow cytometry using FITC-labeled anti-rabbit Fc antibody. A total of 5 × 10^5^ platelets were incubated with wild-type RBD-rFc or RBD-RGE-rFc (RBD with RGE mutation). (G) RBD binding to integrin β3 knockout, and wild-type BALB/c mice platelets were analyzed by flow cytometry using Alexa Fluor 647-labeled anti-RBD mAb 4H12. All the data are displayed as means ± SEM. Two-way ANOVA Tukey’s multiple comparisons test was applied to evaluate the difference between groups. *P < 0.05, **P < 0.01, ***P < 0.0001. (J) Direct binding analysis of labeled RBD as a function of integrin αIIbβ3 concentration in vitro utilizing normalized mean fluorescence anisotropy of Alexa Fluor 488 in binding buffer. (K) Direct binding kinetics characterization of spike-αIIbβ3 using BLI in binding buffer (20 mM tris [pH 7.4], 137 mM NaCl, 1 mM MgCl_2_, 1 mM MnCl_2_, 1 mM CaCl_2_, and 50% glycerol) plus 1X Octet Kinetics Buffer in a concentration range from 68 to 512 nM (red sensograms) at 25 °C. Mean control sensogram (black) is 2% BSA in binding buffer.

To investigate how the RBD induced platelet clearance in vivo, we assessed its binding to platelets by flow cytometry. We found that RBD binds to both human and mouse platelets (Fig. 2A to I) and that RBD (20, 50, 100, and 200 μg/ml) could bind to human platelets in a dose-dependent manner. Platelet expression of ACE2 has been a contentious topic. We assessed ACE2 presence on human platelets with 5 different anti-ACE2 antibodies via western blot. Our results show positive bands in nonreducing protein samples with an approximate molecular weight of 250 kDa (Fig. S2), indicating that dimeric ACE2 may distribute on the human platelets. However, we still cannot exclude the possibility that these antibodies may cross-react with other mimotopes.

The RBD contains an RGD (arginine-glycine-aspartate) tripeptide motif that is known to bind integrins, which are highly expressed on platelets [50,58]. Interestingly, we found that the RBD binds to β3^−/−^ mouse platelets significantly less relative to wild-type platelets (Fig. 2G). Preincubation of platelets with β3 antagonist eptifibatide or anti-β3 monoclonal antibody (mAb) M1 blocks RBD binding to human and murine platelets, respectively (Fig. 2C and H). We then mutated the RGD motif to RGE (arginine-glycine-glutamate), which is known to abrogate integrin binding, and found that the RGD/E mutation reduced RBD binding to platelets (Fig. 2D and I). We further examined direct binding of RBD with recombinant αIIbβ3 in vitro using a fluorescence anisotropy assay and quantified the dissociation constant (*K*_d_) of (96 ± 8) nM and a binding stoichiometry value of 1.3 at 25 °C (Fig. 2J). Also, we examined direct binding kinetics of the full-length spike protein to quantify the affinity parameters of spike–αIIbβ3. Our global association and dissociation analyses of the obtained sensograms quantifies the *k_on_* and *k_off_* rates (Table S1) with a *K*_d_ value of (47 ± 2) nM for recombinant spike and purified αIIbβ3 binding at 25 °C (Fig. 2K). Moreover, our calorimetry study confirmed a sigmoidal thermogram, evident for the direct interactions of spike–αIIbβ3 integrin at 25 °C (Fig. S3). Taken together, the spike protein and RBD bind platelets partially via the RGD motif–αIIbβ3 integrin interaction.

### The RBD induces platelet activation and potentiates platelet aggregation in vitro

**Fig. 3.**
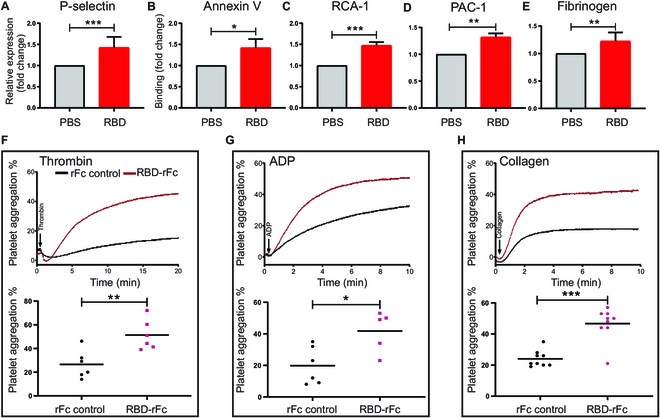
The RBD induces human platelet activation and potentiates platelet aggregation in vitro*.* (A to E) Human platelet activation (P-selection expression; PAC-1, fibrinogen binding), apoptosis (Annexin V binding) and desialylation (RCA-1 binding) were detected via flow cytometry. RBD without tag: 50 μg/ml, *n* = 3. All flow cytometry data are expressed as fold change from control group. (F to H) Human gel-filtered platelet (preincubated with 200 μg/ml RBD-rFc) aggregation was stimulated by 0.02 U/ml thrombin, or 2 μM ADP with fibrinogen, or 2 μg/ml collagen. *n* = 5 to 9. All the data were expressed as means ± SEM (***P < 0.001, **P < 0.01, *P < 0.05).

We hypothesized the RBD binding to platelets may directly induce platelet clearance by promoting activation and aggregation. To investigate this, we incubated human platelets with RBD and evaluated a panel of platelet activation markers by flow cytometry. These include P-selectin, the prototypical marker of platelet degranulation; Annexin V, to detect exposed phosphatidylserine involved in cell-based thrombin generation [59,60]; platelet activation complex-1 (PAC-1) mAb/fibrinogen binding; and *Ricinus communis* agglutinin-1 (RCA-1) binding, an emerging platelet activation marker that detects desialylation [17,61], which has been demonstrated by us and others to trigger liver-mediated platelet clearance [17,22,62]. We found the RBD increases P-selection expression, Annexin V binding, RCA-1 binding, and PAC-1/fibrinogen binding to platelets (RBD versus control, *P < 0.05, Fig. 3A to E and Fig. S4). We also demonstrated that the RBD potentiates platelet aggregation in human platelets with low-dose thrombin, adenosine diphosphate (ADP), and collagen (Fig. 3F to H and Fig. S5A and S5B). As expected, the RBD without a tag also potentiated platelet aggregation (Figs. S5C and D and S6), and the RBD-induced platelet activation and enhanced aggregation were attenuated by β3 antagonist eptifibatide (Fig. S5E to G).

### The κ and δ variants have greater potential to bind, activate, and aggregate platelets

**Fig. 4.**
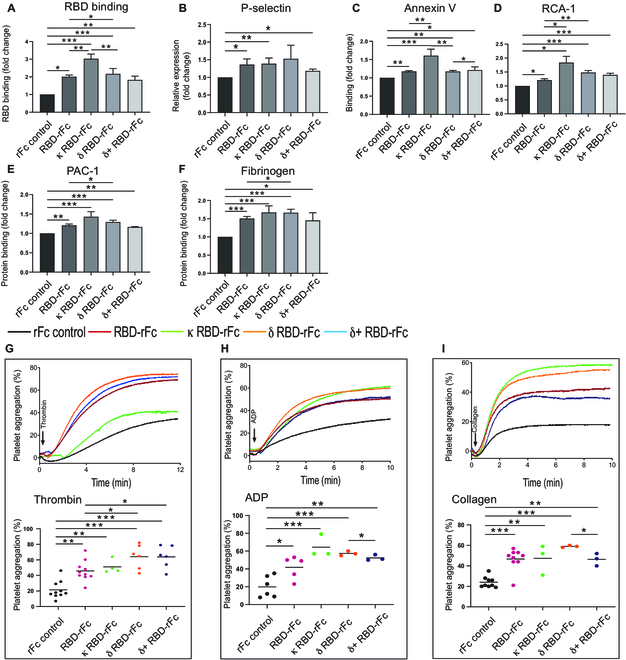
The κ, δ, and δ+ variants have enhanced platelet binding, activation, and potentiation of platelet aggregation. (A) The binding of RBD-rFc variants (200 μg/ml) to human healthy donors’ platelets was analyzed by flow cytometry using FITC-labeled anti-rabbit IgG (Fc specific). (B to F) Human platelet activation (P-selection expression/PAC-1 or fibrinogen binding), apoptosis (Annexin V binding), and desialylation (RCA-1 binding) were detected via flow cytometry, *n* = 5 to 10. (G to I) Human gel-filtered platelet (preincubated with 200 μg/ml RBD-rFc or RBD-rFc variants) aggregation was stimulated by 0.02 U/ml thrombin, or 2 μM ADP with addition of fibrinogen or 2 μg/ml collagen. All the data were expressed as means ± SEM. *P < 0.05, **P < 0.01, ***P < 0.001.

We constructed 3 RBD mutants of B.1.617.1 (Kappa strain), B.1.617.2 (Delta strain), and B.1.617.2.1 (Delta Plus (+) strain) and compared their effect on platelet activation and aggregation relative to wild-type RBD. Flow cytometry results showed that all 3 RBD variants bind to human platelets (Fig. 4A) and induce platelet activation (Fig. 4B to F). Furthermore, we found trending evidence for more pronounced platelet activation (Fig. 4B to F) and platelet aggregation potentiation (Fig. 4G to I) induced by the κ and δ variants, which was greatest in the κ variant. Together, these data indicate that the κ and δ variants induce stronger platelet activation, which is consistent with clinical reports of severe thrombosis in patients infected with these variants [11,63,64].

### Novel anti-RBD mAbs inhibit RBD-induced activation, aggregation, and clearance in vivo

**Fig. 5.**
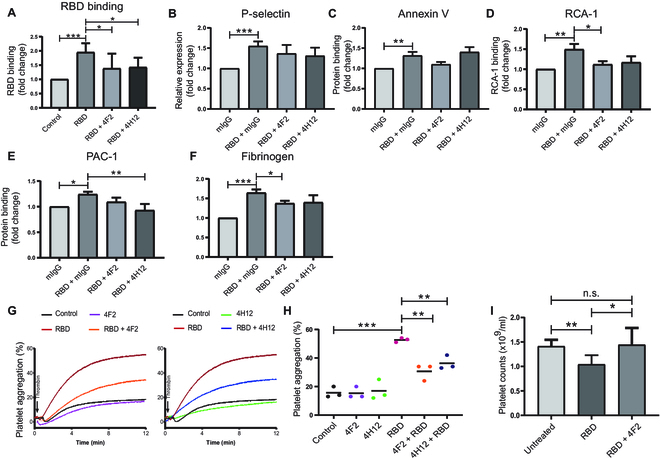
The novel anti-RBD mAbs 4F2 and 4H12 inhibit RBD-induced platelet activation and RBD-potentiated platelet aggregation. (A) The inhibitive effect of anti-RBD mAbs (50 μg/ml) on RBD-rFc (200 μg/ml) binding to human washed platelets was analyzed by flow cytometry using FITC-labeled anti-rabbit IgG (Fc specific). (B to F) The inhibitive effect of anti-RBD mAbs 4F2 and 4H12 (50 μg/ml) on RBD-induced human platelet activation (P-selection expression/PAC-1 or fibrinogen binding), apoptosis (Annexin V binding), and desialylation (RCA-1 binding) was detected via flow cytometry. (G and H) Human gel-filtered platelet (preincubated with rFC control, 200 μg/ml RBD-rFc, or 50 μg/ml 4F2 + 200 μg/ml RBD-rFc) aggregation was stimulated by 0.02 U/ml thrombin. (I) CD1 mouse platelets were counted at 0 h (Untreated) and 3 h after I.V. injection with RBD-rFc or RBD-rFc + 4F2 (RBD-rFc incubated with 4F2 before injection, *n* = 3). RBD-rFc: 1 μg/g, 4F2: 1 μg/g. All data are expressed as means ± SEM. n.s. denotes no significance. *P < 0.05, **P < 0.01, ***P < 0.001.

We generated a panel of anti-RBD mouse mAbs using the hybridoma technique (Table). We selected 4F2 and 4H12 for further study based on their strong inhibition of the RBD–ACE2 interaction. Interestingly, 4F2 and 4H12 also inhibited RBD binding to human platelets (Fig. 5A) and suppressed RBD-induced platelet clearance in vivo (Fig. 5I). 4F2 and 4H12 also alleviated RBD-induced human platelet activation (Fig. 5B to F) and RBD-potentiated platelet aggregation in vitro (Fig. 5G and H), while the mAbs themselves did not induce platelet activation/aggregation. 4F2 and 4H12 could still recognize and neutralize the κ variant, inhibiting its binding to human platelets (Fig. S7A) and alleviating its platelet activation in vitro (Fig. S7B to D). The other 3 anti-RBD mAbs 4H10/2B5/4E10 also alleviated RBD-induced human platelet activation in vitro (Table).

**Table.** Characterization of our novel anti-RBD antibodies on RBD-induced platelet activation.

**Clone name**	**IgG subtype**	**Inhibit RBD–ACE2 binding**	**Inhibit RBD-PLT binding**	**Inhibit P-selectin**	**Inhibit** **PAC-1**	**Inhibit fibrinogen**
4F2	IgG 1	Strong	Yes	Yes (*)	Yes	Yes (*)
4H10	IgG 2a	Weak	Yes	Yes	Yes (*)	Yes (**)
2B5	IgG 2a	Weak	Yes	Yes	Yes (*)	Yes (*)
4E10	IgG 2b	Weak	Yes	Yes	Yes	Yes
4H12	IgG 1	Strong	Yes	Yes	Yes (**)	Yes

*P < 0.05, **P < 0.01, RBD + anti-RBD mAb vs. RBD + mIgG control.

### Antibodies 4F2 and 4H12 dose-dependently inhibit SARS-CoV-2 infection of Vero E6 cells

**Fig. 6.**
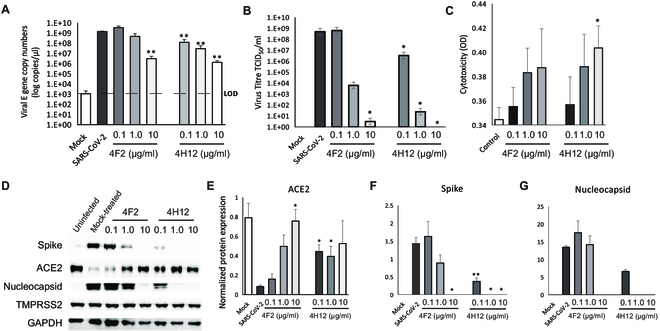
Anti-RBD mAbs 4F2 and 4H12 inhibit SARS-CoV-2 infection in a dose-dependent manner. (A) Different concentrations of anti-RBD mAb 4F2 or 4H12 were mixed with MOI 2 of SARS-CoV-2 for 30 min and then added to the culture medium of Vero E6 cells (the inoculum was not removed). Vero E6 cells were recovered 15 h postinfection and viral RNA was assayed by RT-qPCR. Dotted horizontal lines indicate the limit of detection (LOD). (B) Virus progeny was evaluated for viable virus in a TCID_50_ assay. (C) The addition of 4F2 or 4H12 was not toxic to the Vero E6 cells. (D) Detection of SARS-CoV-2 entry proteins and viral proteins in cells lysate by western blot. Glyceraldehyde-3-phosphate dehydrogenase was used as a loading control. (E to G) Densitometric analysis of protein bands. Data are represented as means ± SEM. *P < 0.05, **P < 0.01, versus SARS-CoV-2 group.

To investigate whether our anti-RBD 4F2 and 4H12 antibodies could interfere with SARS-CoV-2 virus infection, we infected Vero E6 cells with SARS-CoV-2/SB2 at a multiplicity of infection (MOI) of 2 [65]. The viral envelope gene was quantified by reverse transcription real-time quantitative polymerase chain reaction (RT-qPCR) assay (Fig. 6A) as a marker for viral replication. SARS-CoV-2 infection of Vero E6 cells was significantly inhibited 15 h after introduction of cells to media preincubated with virus and either 4F2 or 4H12 dose-dependently (Fig. 6A). In line with reduced viral load, we observed a significant reduction in SARS-CoV-2 titers in 4F2/4H12 pretreated Vero E6 cells (*P < 0.05; Fig. 6B) in a dose-dependent manner to near undetectable levels. Cytotoxicity studies revealed that neither 4F2 nor 4H12 was toxic to Vero E6 cells (Fig. 6C). Next, we examined the expression of viral and entry proteins. Interestingly, we saw 4F2 and 4H12 dose-dependently decreased spike and nucleocapsid protein expression but increased/regained ACE2 protein expression (Fig. 6D to G), which is consistent with the earlier reports that SARS-CoV-2 binding leads to ACE2–SARS-CoV-2 complex internalization and lysosomal degradation [66,67]. As expected, transmembrane protease serine 2 expression was independent of SARS-CoV-2 virus infection and 4F2 or 4H12 treatment (Fig. 6D). Collectively, these data demonstrate that 4F2 and 4H12 can inhibit SARS-CoV-2 virus infection and provide support for the study of anti-RBD therapies to treat COVID-19.

## Discussion

COVID-19 continues as a global health threat, in part due to the constant emergence of novel variants. COVID-19 patients face the risk of life-threatening thrombotic events including heart attack and stroke; however, the mechanism remains inadequately understood. We herein demonstrated that the SARS-CoV-2 spike protein RBD causes significant platelet clearance in vivo. Further investigation revealed the RBD bound platelets partially via an interaction between its RGD motif and the αIIbβ3 integrin. RBD–platelet binding could induce platelet activation and potentiate platelet aggregation, which was amplified in the Delta and Kappa variants. Notably, the RBD also induced platelet desialylation, suggesting liver-mediated platelet clearance. Lastly, we generated a panel of novel anti-RBD mAbs, including 4F2 and 4H12, that inhibited RBD-induced platelet activation and clearance and also attenuated SARS-CoV-2 infection of Vero E6 cells. These findings reveal a novel mechanism of RBD-mediated thrombosis and platelet clearance with implications for both COVID-19 and VITT and also provide justification for the use of anti-RBD mAbs for treating COVID-19.

Several viruses are reported to interact with integrins [68,69]. More recently, the RGD motif in SARS-CoV-2 RBD is reported to interact with integrins αVβ3, αVβ6, and α5β1 [53–55]. In this report, our data highlights the virus RBD also binds to the αIIbβ3 integrin on platelets via its RGD motif. RBD platelet binding was reduced with αIIbβ3 antagonists eptifibatide and mAb M1, confirming its binding site within the αIIbβ3 ligand-binding pocket. Although structural studies suggest steric hindrance of the RGD motif in the full-length spike protein conformation, it is possible that RGD exposure increases with dynamic structural changes. We propose that thiol-isomerase activity of the β integrin PSI domain may help prime the spike protein for an integrin interaction, especially given its cysteine-rich nature [58,70]. Interestingly, however, we found the RBD–platelet interaction could not be completely inhibited by either αIIbβ3 antagonists or in β3^−/−^ mice, indicating additional receptors are/may be involved. Our western blot showed positive bands for ACE2 in nonreducing protein samples at approximately 250 kDa, suggesting dimeric ACE2 may distribute on human platelets. However, we interpret these results cautiously due to the possibility of antibody cross-reactivity. Binding to additional candidate receptors including c-mpl and CD147 are worth future investigation [25,44,71].

Platelet hyperactivation is well reported in COVID-19. Immune dysregulation such as cytokine storm is an indirect mechanism that is thought to, at least partially, contribute to platelet activation in COVID-19. Our study describes a direct mechanism of RBD-induced platelet activation. Although the mechanism of platelet activation is currently unclear, interactions with ACE2, toll-like receptors, and other binding partners may synergistically contribute to this event [25]. It is also possible that the RGD motif in the RBD contributes to platelet activation, particularly in a multivalent and full-length spike protein conformation. Given the high copy number of spike protein on the virus surface, we cannot exclude the virus; otherwise, this may bridge multiple platelets via RBD–αIIbβ3 interaction, causing platelet aggregation and contributing to microthrombi formation in COVID-19.

COVID-19 patients also exhibit hypercoagulability, yet the SARS-CoV-2 virus does not appear to have intrinsic procoagulant effects [72]. Platelet-monocyte aggregates bearing tissue factor are reported to drive coagulation in COVID-19 [28]. We show the RBD induces P-selectin expression, which promotes the formation of tissue-factor-bearing platelet-monocyte and other leukocyte aggregates. The RBD also induced platelet phosphatidylserine exposure, which harbors coagulation factors and promotes coagulation via cell-based thrombin generation [13,59,70]. Subsequent thrombin may feedback to further activate platelets, creating a vicious positive feedback loop. Taken together, the RBD may directly activate platelets and indirectly orchestrate hypercoagulation to synergistically contribute to thrombotic incidences including deep vein thrombosis, pulmonary emboli, lung microcirculatory thrombi, and arterial thrombosis such as stroke. Thus, although anticoagulant therapy has been a primary treatment against COVID-19 [73], our work provides strong rationale for further investigation of antiplatelet therapy as a potential upstream target.

The Delta (L452R and T478K) and Kappa (L452R and E484Q) variants each bear 2 mutations in the RBD. Our data shows that the Delta and Kappa RBD variants bound to platelets and induced activation and potentiated platelet aggregation significantly more than the original strain. It is possible that these mutations in the RBD may lead to structural alterations that enhance exposure of the RGD integrin-binding motif to promote its interaction with platelets. Recently, Omicron has succeeded as the most common variant and has 15 mutations in RBD region and ~32 mutations in the spike protein. Although these structural changes bolster affinity for the ACE2 receptor [74], the effect on its accessibility for the αΙΙbβ3 integrin is unclear and is worth further investigation.

The prevailing view for VITT has been that anti-RBD antibodies cross-react with platelet factor 4 to create a large immunogenic complex capable of inducing platelet activation via the FcγRIIA [34,75]. This mechanism is reminiscent of heparin-induced thrombocytopenia but occurs in the absence of heparin. Our finding of direct RBD-induced platelet activation may contribute by providing the initial wave of platelet factor 4 release. We also cannot exclude the possibility that administration of the RBD-containing spike protein in adenovirus-based vaccines directly causes VITT. Indeed, our data shows that RBD injection over a concentration threshold could elicit significant platelet clearance. Given the variable RBD concentration derived from DNA/RNA COVID-19 vaccines, it is possible that high RBD concentrations from vaccination may account for some incidences of platelet clearance or even VITT. Thus, future research investigating the correlation between plasma RBD concentration and incidence of VITT merits further study.

Our group previously reported platelet desialylation as a novel mechanism of liver-mediated platelet clearance [14,17,61]. Since the RBD induced significant platelet desialylation, we suspect that the liver may be a key site of RBD-induced platelet clearance. The effect of excessive hepatocellular platelet clearance on liver function is unclear. However, liver injury is reported in both COVID-19 [76] and VITT [77]. Given the importance of the hepatocytes in the production of coagulation factors, an altered function may contribute to coagulopathy, a common symptom in advanced COVID-19 [78]. Furthermore, hepatocytes are also key producers of the platelet regulator thrombopoietin [79,80], thus, hepatocellular dysfunction may impair platelet production and exacerbate thrombocytopenia. On the other hand, our group has also discovered immunosuppression linked with liver-mediated platelet clearance (manuscript submitted to *Nature Communications*). Whether RBD-mediated platelet clearance in the liver can modulate immune response [81–85] or down-regulate coagulation factor and thrombopoietin production requires further investigation.

We found that RBD-induced platelet clearance in vivo and platelet activation and aggregation in vitro were susceptible to the therapeutic effects of our novel mAbs F42 and 4H12. Moreover, 4F2 and 4H12 also demonstrated the capacity to neutralize the RBD–ACE2 interaction. To our knowledge, this is the first reported therapeutic agent capable of inhibiting SARS-CoV-2 infection, thrombosis, and thrombocytopenia.

In summary, our data reveal that the RBD directly binds to platelets and that this binding is partially mediated by RGD–integrin αIIbβ3 interaction. RBD binding causes platelet activation and clearance, which provide new insights into the mechanisms of thrombosis and thrombocytopenia that are observed in COVID-19 and VITT. We also cannot exclude that this interaction may contribute to the virus internalization into platelets, which may contribute to immune evasion and hematological dissemination to new sites of infection. Furthermore, our newly developed anti-RBD mAbs 4F2 and 4H12 can ameliorate RBD-induced platelet activation/clearance and therefore may have the potential not only for diagnosis of SARS-CoV-2 virus antigen but importantly also for treatment of COVID-19 and its complications.

## Materials and Methods

### Blood collection and platelet isolation

All blood collection procedures were approved by the Research Ethics Board of St. Michael’s Hospital (Toronto, ON, Canada) and carried out as previously described [15,58,86]. Venous blood samples from healthy human donors were collected with informed consent into BD Vacutainer plastic blood collection tubes with 3.2% sodium citrate. Mice were anesthetized using 2.5% avertin via intraperitoneal injection, and whole blood was collected into 3.2% sodium citrate via retro-orbital bleeding. Platelet-rich plasma (PRP) was separated by centrifugation (300×g, 10 min). Platelets were washed (1,050×g, 15 min with 60 nM prostaglandin E1 [P5515, Sigma]) in modified Tyrode’s buffer (134 mM NaCl, 2.9 mM KCl, 0.34 mM Na_2_HPO_4_, 12 mM NaHCO_3_, 20 mM Hepes, 1 mM MgCl_2_, and 5 mM glucose, pH 7.35). Then, platelets were counted with a Z2 Series Coulter Counter (Beckman Coulter, USA).

### Mice

Integrin β3^−/−^ mice were kindly provided by Dr. Richard O. Hynes from the Massachusetts Institute of Technology and further backcrossed with BALB/c mice [87,88]. Six-week-old female BALB/c mice and CD1 mice were purchased from Charles River Canada. All mice were housed and bred in the St. Michael’s Hospital Research Vivarium. The animal procedures were approved by the Animal Care Committee and in compliance with the Guidelines of the Canadian Council of Animal Care.

### Cell lines

Vero E6 cells (African green monkey cells; CRL-1586, American Type Culture Collection) were maintained in Dulbecco’s modified Eagle medium (DMEM) supplemented with 10% fetal bovine serum (Sigma-Aldrich) and 1 × l-glutamine and penicillin/streptomycin (Corning).

### SARS-CoV-2 virus

SARS-CoV-2 SB3-TYAGNC was generously provided by Dr. Samira Murabeka from Sunnybrook Research Institute, Toronto. The virus was isolated from a nasopharyngeal sample of a patient in Canada. Supernatant from the cell lysate was used to determine virus titers (50% tissue culture infectious dose [TCID_50_]/ml) according to the Spearman and Karber method as outlined previously [65].

### SARS-CoV-2 virus infection

All work involving live SARS-CoV-2 was performed in the Combined Containment Level 3 Unit (C-CL3 Unit) of the Temerty Faculty of Medicine at the University of Toronto in accordance with institutional biosafety requirements. Vero E6 cells were seeded in 48-well plates (5 × 10^4^ cells per well) (Sarstedt) in DMEM containing 10% fetal bovine serum. Twenty-four hours postseeding, different concentrations of our newly developed antibody 4F2/4H12 were mixed with SARS-CoV-2 at an MOI of 2 in a final volume of 100 μl of DMEM per well at 37 °C. After 30 min, Vero E6 cells were infected with mixes containing antibody 4F2/4H12 and SARS-CoV-2 (the inoculum was not removed). Fifteen hours postinfection, supernatants were removed, and cells were recovered with viral RNA quantified by RT-qPCR.

### RT-qPCR

Samples were extracted using the QiaAmp Viral mini kit (52906, QIAGEN) according to the manufacturer’s instructions. RT-qPCR was performed using E-gene SARS-CoV-2 following guidelines by the World Health Organization (https://www.who.int). The RT-PCR assays were performed by using Luna Universal qPCR Master Mix (E3005L, New England Biolabs) based on the manufacturer’s instructions. Two separate gene targets were employed for detecting the envelope (E) gene. The E-gene CAGGTACGTTAATAGTTAATAGCGT and ATATTGCAGCAGTACGCACACA were used as forward and reverse primers. qPCR was performed on an Applied Biosystems QuantStudio 7 Flex Real-Time PCR System. The reaction conditions were 1 cycle of denaturation (60 °C for 10 min, then 95 °C for 2 min), followed by 40 amplification cycles (95 °C for 10 s and 60 °C for 15 s). QuantStudio Real-Time PCR software was used for analysis.

### Construction of RBD recombinant proteins

SARS-Cov-2 spike protein subunit 1 (RBD residues, V327 to T531) was cloned in AbVec2.0-IGHG1 plasmid (80795, Addgene) and fused with glycine serine linker (Gly4 S)1 and mouse/rabbit Fc-tag on C-terminus. An N-terminal 10× His-tag was fused with (G3S1)1, TEV cleavage site and RBD residue V327 to T531. Unless stated otherwise, the RBD and full spike protein sequences used in this study were derived from the original SARS-CoV-2 virus. Kappa variant (L452R, E484Q)/Delta variant (L452R, T478K)/Delta+ variant (K417N, L452R, T478K) RBD rabbit Fc-tagged constructs and RBD (RGE mutation) rabbit Fc-tagged mutant (D405E) were generated using an In-Fusion Cloning Kit (Takara Bio, Inc.). RBD without a tag was generated after TEV protease cleavage using an N-terminal His-tag RBD construct. These RBD constructs were transfected in Expi293F cells (3 × 10^6^ viable cells/ml) in Expi293 Expression Medium (Thermo Fisher Scientific) using FectoPRO transfection reagent (Polyplus-transfection) at 37 °C, 8% CO_2_ under 120 rpm shaking conditions for 3 to 4 d. RBD proteins were purified using Protein A agarose or HisPur nickel nitrilotriacetic acid resin (Thermo Fisher Scientific). Proteins were buffer exchanged with phosphate-buffered saline (PBS) (10 mM sodium phosphate buffer, pH 7.4, 2.7 mM KCl, and 137 mM NaCl) and stored at −80 °C until use.

### Development and purification of mAbs against RBD

Female BALB/c mice 6 to 8 weeks old were immunized with 30 to 50 μg of RBD recombinant protein mixed with TiterMax (Sigma-Aldrich, Canada) 3 times as we previously described [17,58,89]. The immunized spleen cells were then fused with mouse myeloma cells (P3X63Ag8.653, American Type Culture Collection), and hybridomas were selected by HAT medium (Sigma-Aldrich, Canada). Positive hybridomas were identified using enzyme-linked immunosorbent assay and subcloned through limit dilution. Hybridomas that secret antibodies were cultured in sera-free medium for large-scale antibody production. The mAbs were isolated/purified using Protein-G agarose beads (Thermo Fisher Scientific).

### RBD injection and platelet count assay

We injected different doses (0.25, 0.5, and 1.0 μg/g) of RBD into CD1 mice via tail vein injection and counted the number of platelets at 0, 1, 3, 8, 24, and 48 h postinjection. CD1 mice were bled via the saphenous vein, and platelet count procedures were performed as we previously described [17,56,57]. Briefly, 10 μl of whole blood per mouse was mixed with 240 μl of PBS-EDTA, spun down (150×g, 3 min), then 50 μl of supernatant was mixed with 10 ml of Isotone II Diluent and platelets were counted with a Z2 Series-Coulter-Counter. We examined the effect of RBD-rFc on platelet counts applying a dose-response assay by quantifying the rate of change in platelet counts as a function of elapsed time and the amount of administered dose. Then, we quantified the effective concentration value for RBD-Fc utilizing a dose-response analysis model in OriginPro (OriginLab, 2016).

### Flow cytometry analysis

For analysis of labeled transfused platelets [17], CD1 mouse platelets were labeled with Far Red dye (Thermo Fisher Scientific) for 30 min at 37 °C and washed with PBS buffer. Labeled platelets (10^8^ counts) were then incubated with RBD for 30 min and then transfused via tail vein into syngeneic mice. Mice were bled at 0 h (baseline, following platelet transfusion), 1, 3, 24 h posttransfusion via retro-orbital bleeding into PBS-EDTA and centrifuged at 150×g, 3 min. The supernatant was then analyzed via flow cytometry to detect the percentage of fluorescent positive platelets. For analysis of platelet activation, human platelet surface P-selectin was detected with anti-human CD62P (P-Selectin) fluorescein isothiocyanate (FITC) antibody (304904, Biolegend); GPIIbIIIa activation was detected with anti-human CD41/CD61 PAC-1 Alexa Fluor 647 antibody (362806, Biolegend) for human platelets. Annexin V, fibrinogen, and RCA-1 binding were detected with Alexa Fluor 647 Annexin V (640912, Biolegend), Alexa Fluor 647 Fibrinogen (F35200, Thermo Fisher Scientific) and fluorescein labeled RCA-1 (FL-1081-5, Vector Labs), respectively, as we previously described [26,61,90–92]. For analysis of RBD binding, RBD (with rabbit Fc tag) binding was detected with anti-rabbit immunoglobulin G (IgG) [Fc specific]-fluorescein antibody (1:500, SAB3700850, Sigma). Anti-RBD antibody 4H12 was labeled with an antibody labeling kit (Cat. No. A20181, ThermoFisher) according to the product instructions and RBD binding was detected using Alexa Fluor 647 4H12 in a dilution of 1:100. A total of 10,000 platelet events were acquired and data were analyzed via FlowJo 7.6 (Becton, Dickinson and Company, NJ, USA).

### Direct binding analysis of RBD-αIIbβ3 in vitro

The recombinant monomeric RBD protein (22 kDa) was expressed and purified. Then, the purification tag was cleaved as described above. The covalent conjugation of the N-terminal RBD monomer to a fluorescein derivative label was performed through an amine-tetrafluorophenyl reaction in PBS at 25 °C (Alexa Fluor 488, Invitrogen). The labeled RBD was further purified and concentrated in the binding buffer (20 mM tris, [pH 7.4], 137 mM NaCl, 1 mM CaCl_2_, 1 mM MgCl_2_, and 1 mM MnCl_2_). We measured the fluorescence anisotropy (*r*) of 5 nM labeled RBD in a titration with purified αIIbβ3 protein (GPIIbIIIa, Innovative Research) in the binding buffer at 25 °C using a Cary Eclipse spectrofluorometer (λ_ex_ = 495 nm and λ_em_ = 517 nm) as previously described [93]. The results from 3 trials were averaged, normalized, and plotted as a function of αIIbβ3 concentration. To quantify the dissociation constant (*K*_d_) values, the binding curve was fit to a 1-site binding function as described elsewhere [94].

### Western blot

Total proteins of platelets were extracted by RIPA Lysis and Extraction Buffer (89900, Thermo Fisher Scientific). The proteins of platelets or Vero E6 cells or purified RBD were separated by 8% bis-tris sodium dodecyl sulfate polyacrylamide gel electrophoresis and transferred to nitrocellulose membranes (1620115, Bio-Rad). The blots were blocked with 5% fat-free milk (sc-2325, Santa Cruz Biotechnology) for 1 h at room temperature and then with an anti-ACE2 antibody (1:1,000, ab15348/ab272500/ab108252, Abcam; MA5-32307, Thermo Fisher Scientific; 21115-1-AP, Proteintech), anti-nucleocapsid antibody (40143-R001, Cell Signaling), anti-spike antibody (ZMS1076, Sigma), anti-transmembrane protease serine 2 antibody (AB92323, Abcam). or anti-glyceraldehyde-3-phosphate dehydrogenase antibody (5174, Cell Signaling) overnight at 4 °C. The membranes were then incubated with horse radish-conjugated goat anti-mouse IgG (1:3,000, 62-6520, Thermo Fisher Scientific) or anti-rabbit IgG (1:10,000, 111-035-003, Jackson ImmunoResearch) for 1.5 h at room temperature and visualized with enhanced chemiluminescence (ECL) substrate (32106, Thermo Fisher Scientific) and Bio-Rad ChemiDoc imaging system. Densitometry was performed using Image Lab software (Bio-Rad).

### Platelet aggregation assay

PRP or human gel-filtered platelets were prepared as we previously described [95–98]. Gel-filtered platelets were isolated from PRP using a Sepharose 2B chromatography column with PIPES buffer. Platelet count was adjusted to 2 × 10^8^ counts/ml and preincubated with 200 μg/ml RBD or RBD variants with or without 50 μg/ml anti-RBD antibodies. Platelet aggregation was induced by 0.02 to 0.04 U/ml thrombin, 2 μM ADP with the addition of fibrinogen, 2 μg/ml collagen with 2 mM CaCl_2_. Platelet aggregation was measured in a Chrono-Log Model 700 aggregometer (Chrono-Log, USA). and data were collected and recorded for at least 8 min by Aggrolink8 software.

### Biolayer interferometry

Recombinant spike protein samples with a C-terminal 8× His-tag were prepared as described in Construction of RBD recombinant proteins. Purified spike protein samples (0.35 μM) were immobilized onto hydrated nickel nitrilotriacetic acid biosensor probes employing an 8-channel Octet RH16 biolayer interferometry instrument and tilted-bottom microplates in the supplied Octet Kinetics Buffer (Sartorius, Germany). Probes were quenched with SuperBlock buffer (Thermo Fisher Scientific) and equilibrated in binding buffer (20 mM tris [pH 7.4], 137 mM NaCl, 1 mM MgCl_2_, 1 mM MnCl_2_, 1 mM CaCl_2_, and 50% [v/v] glycerol) plus 1X Octet Kinetics Buffer shaken at 1,000 rpm to obtain a baseline response for 3 min at 25 °C. The wavelength shifts corresponding to the association rates (*k_on_*) were measured by immersing the loaded biosensors into wells containing platelet-derived purified human αIIbβ3 protein (Innovative Research) in binding buffer at a concentration range from zero to 0.51 μM. The dissociation rates (*k_off_*) were measured by transferring the loaded biosensors into wells containing binding buffer. The effect of binding buffer on the threshold of detection was optimized, and raw data were subtracted from a reference loaded biosensor in the binding buffer. A triplicated sample of 0.3 mM bovine serum albumin (2% BSA) in binding buffer was used as a nonspecific binding control. All concentrations were verified by using NanoDrop (Thermo Fisher Scientific ND-One). The acquired wavelength shifts from a minimum of 3 trials were fit to a global binding model and analyzed to quantify binding parameters using the supplied Octet software package and GraphPad Prism.

### Isothermal titration calorimetry

Purified spike protein samples (12 μM) were titrated in 1 μM purified human αIIbβ3 integrin samples, and the heat of binding was measured utilizing an isothermal titration calorimetry (ITC) instrument (Microcal ITC-200). Samples were degassed before use with a MicroCal Thermo Vac unit for 5 min. Titrations were performed with αIIbβ3 integrin in the cell and recombinant spike protein serving as the titrant in the syringe. All experiments were performed in 20 mM tris (pH 7.4), 100 mM NaCl, 1 mM CaCl_2_, 1 mM MnCl_2_, 1 mM MgCl_2_, 0.05% NaN_3_, and 10% (v/v) glycerol at 25 °C. Each trial consisted of an initial delay of 60 s, first injection of 0.2 μl and 300-s delay. A subsequent 18 injections were 2 μl, spaced at 300-s intervals. The first point was removed from all data sets due to different injection volume and delay parameters. The acquired ITC data were subtracted from a titration of spike protein in a blank buffer control. The analyzed thermogram was fit to a 1-site binding model using the manufacturer provided Origin 7 software.

### Statistical analysis

All the data were analyzed using GraphPad Prism 8.0 (GraphPad Software Inc., USA), and presented as mean ± standard error of mean (SEM). Statistical significance was determined using an unpaired Student *t* test (2-tailed) for comparisons between 2 groups. One-way analysis of variance (ANOVA) followed by Dunnett’s and Tukey’s multiple comparisons tests were used to compare the statistical significances among multiple groups. A *P* value < 0.05 was considered statistically significant.

## Data Availability

The data of this study are available from the first and corresponding authors upon request.

## Supplementary Materials

Supplementary Materials Fig. S1*.* The RBD can induce platelet clearance in vivo*.* Fig. S2. Dimeric ACE2 may distribute on human platelets. Fig. S3. ITC thermograms demonstrate full-length spike protein binding to αIIbβ3 integrin. Fig. S4. The RBD can induce human platelet activation. Fig. S5. The RBD can potentiate human gel-filtered platelet aggregation. Fig. S6. The RBD potentiate human PRP aggregation in vitro. Fig. S7. 4F2 and 4H12 alleviate κ variant RBD-induced human platelet activation in vitro. Table S1. Direct interaction kinetics parameters for biolayer interferometry analyses of full-length spike protein and purified human αIIbβ3 in vitro.
