# Supplementary material for: SARS-CoV-2 RBD and Its Variants Can Induce Platelet Activation and Clearance: Implications for Antibody Therapy and Vaccinations against COVID-19
Source: Research (Wash D C). 2023 Apr 24;6:0124. doi: 10.34133/research.0124 (PMC10202384; doi:10.34133/research.0124)
Supplement: Supplementary Materials — Fig. S1. The RBD can induce platelet clearance in vivo. Fig. S2. Dimeric ACE2 may distribute on human platelets. Fig. S3. ITC thermograms demonstrate full-length spike protein binding to αIIbβ3 integrin. Fig. S4. The RBD can induce human platelet activation. Fig. S5. The RBD can potentiate human gel-filtered platelet aggregation. Fig. S6. The RBD potentiate human PRP aggregation in vitro. Fig. S7. 4F2 and 4H12 alleviate κ variant RBD-induced human platelet activation in vitro. Table S1. Direct interaction kinetics parameters for biolayer interferometry analyses of full-length spike protein and purified human αIIbβ3 in vitro. [file research.0124.f1.docx]

**Supplementary Figure 1:**

**
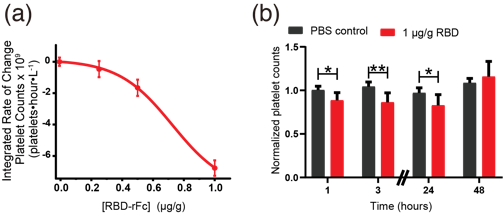
**

**Supplementary Figure 1*.* The RBD can induce platelet clearance *in vivo.*** (**a**) The dose-response analysis of the effect of RBD-Fc on mice platelet clearance in vivo (data set was obtained from Fig. 1a). Shown is the integrated rate of change of platelet counts in 48h post-injection as a function of administered doses. **(b)** CD1 mouse platelets were counted at 1 h and 3 h after individual I.V. injection with a PBS control and RBD without rFc tag (PBS, n = 6; 1 μg/g RBD, n = 4). All the data were expressed as mean ± SEM. Two-way ANOVA followed by Tukey’s multiples comparison test was applied to evaluate the difference between RBD group and control group. **p* < 0.05, ***p* < 0.01, versus control at the same time point.

**Supplementary Figure 2:**

**Supplementary Figure 2. Dimeric ACE2 may distribute on human platelets.** The expression of ACE2 in resting platelets prepared from healthy donors or in VeroE6 cells was analyzed by western blotting using anti-ACE2 monoclonal antibody (ab272500/ab108252, Abcam; ThermoFisher, MA5-32307) and anti-ACE2 polyclonal antibody (ab15348, Abcam; 21115-1-AP, Proteintech). Lanes denoted r for reducing samples and n.r for non-reducing samples.

**Supplementary Figure 3:**

**Supplementary Figure 3. ITC thermograms demonstrate full-length spike protein binding to αIIbβ3 integrin.** **(a)** Displays the titration data for the heat of binding evolved from each injection of recombinant full-length spike protein into purified human αIIbβ3 integrin; **(b)** Demonstrates the integrated heat plot after subtracting for the heat of dilution of spike protein in a control blank buffer. The sigmoidal curve represents the nonlinear fit to a one-site binding model. Titrations were performed at 25 °C in 20 mM Tris (pH 7.4), 100 mM NaCl, 1 mM CaCl_2_, 1 mM MnCl_2_, 1 mM MgCl_2_, 0.05% (w/v) NaN_3_, 10% (v/v) glycerol.

**Supplementary Figure 4:**

**Supplementary Figure 4. The RBD can induce human platelet activation.** **(a-e)** Representative histograms show gated platelet counts versus arbitrary units of fluorescence intensity of FITC for P-selection expression, Annexin V, RCA-1, and Alexa 647 for PAC-1 and fibrinogen binding detected using flow cytometry.

**Supplementary Figure 5:**


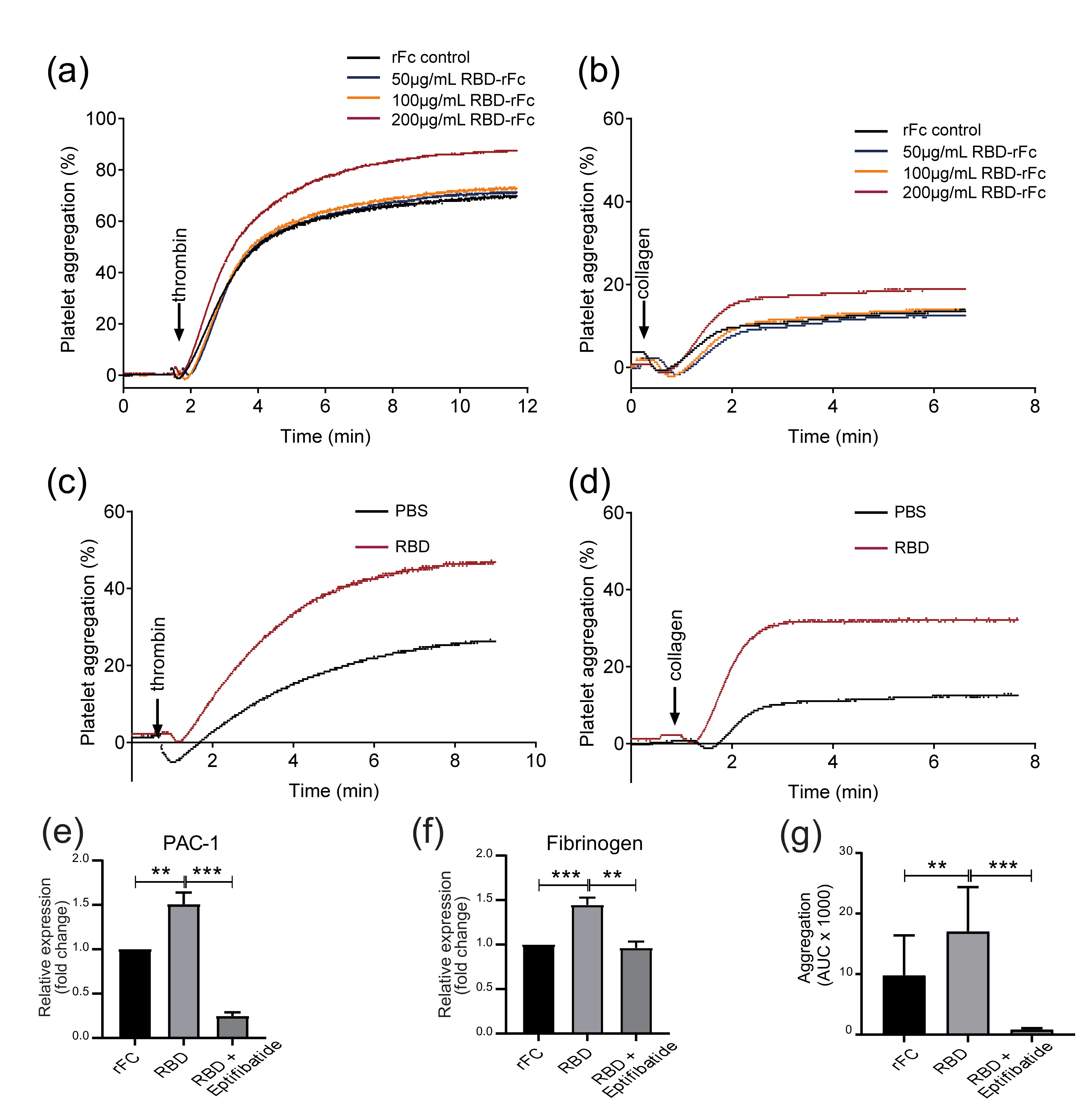


**Supplementary Figure 5. The RBD can potentiate human gel-filtered platelet aggregation. (a-b)** Human gel-filtered platelet (preincubated with 50, 100, 200 μg/mL RBD-rFc) aggregation was stimulated by 0.02 U/mL thrombin and 2 μg/mL collagen, separately. **(c-d)** Human gel-filtered platelet (preincubated with 200 μg/mL RBD without tag) aggregation was stimulated by 0.02 U/mL thrombin and 2 μg/mL collagen, separately. **(e, f)** RBD-induced (50 μg/mL) human platelet activation (PAC-1 or fibrinogen binding) and enhanced aggregation **(g)** was inhibited by β3 antagonist Eptifibatide.

**Supplementary Figure 6:**

**Supplementary Figure 6. The RBD potentiate human PRP aggregation *in vitro*. (a-b)** Human PRP (preincubated with 200 μg/mL RBD without tag) aggregation was stimulated by 0.5 μM ADP or 0.5 μg/mL collagen. Platelet aggregation was measured for at least 8 minutes. All the data were expressed as mean ± SEM (****p* < 0.001, **p* < 0.05).

**Supplementary Figure 7:**

**Supplementary Figure 7. 4F2 and 4H12 alleviate κ variant RBD-induced human platelet activation *in vitro***. **(a)** The inhibitory effect of anti-RBD mAbs (50 μg/mL) on κ variant RBD-rFc (200 μg/mL) binding to human platelets was analyzed by flow cytometry using FITC-labeled anti-rabbit IgG (Fc specific). **(b-d)** The inhibitory effect of anti-RBD mAbs on κ variant RBD-induced human platelet activation (PAC-1/fibrinogen binding) or desialylation (RCA-1 binding) was detected via flow cytometry. All the data were analyzed as mean ± SEM.

**Supplementary Table 1. Direct interaction kinetics parameters for biolayer interferometry analyses of full-length spike protein and purified human αIIbβ3 *in vitro*.**

| [αIIbβ3]  (nM) | *k_on_*  (M^-1^s^-1^) | *k_off_*  (s^-1^) |
| --- | --- | --- |
| 512 | 4.4 × 10^4^ ± 302.3 | 2.0 × 10^-3^ ± 3.0 × 10^-5^ |
| 257 | 2.1 × 10^4^ ± 114.7 | 9.6 × 10^-4^ ± 1.8 × 10^-5^ |
| 110 | 8.9 × 10^3^ ± 63.2 | 4.2 × 10^-4^ ± 3.2 × 10^-6^ |
| 93 | 7.3 × 10^3^ ± 66.7 | 3.4 × 10^-4^ ± 5.1 × 10^-6^ |
| 68 | 5.5 × 10^3^ ± 36.9 | 2.5 × 10^-4^ ± 4.5× 10^-6^ |

The *k_on_* and *k_off_* parameters denote association rate and dissociation rate respectively from a global fit to triplicated experiments.
